# P-1795. Impact of Molecular Diagnostics on Treatment Decisions in General Infectious Diseases Consultative Services: A Call for Laboratory Stewardship

**DOI:** 10.1093/ofid/ofaf695.1964

**Published:** 2026-01-11

**Authors:** Shaurya Sharma, Jennifer Lee, Tamara Nawar, John Quale

**Affiliations:** SUNY Downstate University of Health Sciences, Brooklyn, NY; NYC Health + Hospitals/Kings County, Brooklyn, New York; NYC Health + Hopitals/Kings County, Brooklyn, New York; NYC Health + Hospitals/Kings County, Brooklyn, New York

## Abstract

**Background:**

Molecular diagnostics are being increasingly utilized to diagnose infectious diseases. Rapid identification of bacteria/mycobacteria in normally-sterile tissues and body fluids can be accomplished by amplification and sequencing of a fragment of the 16S ribosomal RNA gene. In addition, metagenomic sequencing of cell-free plasma (MSCFP) can identify a large number of bacterial and non-bacterial pathogens, and has been found to be particularly useful in immunocompromised patients.

**Methods:**

The NYC Health + Hospitals system comprises 11 acute care hospitals of which none are designated transplant or cancer centers. During our study, we reviewed patients whose samples were sent for 16S ribosomal sequencing (16SRS) and MSCFP between May 2023 and April 2025, with a particular focus on the impact of the test results on changes in treatment.

**Results:**

59 patients had samples sent for 16SRS; frequent specimen sources included bone (n=15), joint (n=13), pulmonary (n=8), abdomen (n=7), brain/spinal fluid (n=6), and heart valve (n=4). Treatment was altered due to the 16SRS results in 10 cases, including two patients with heart valve samples (P=0.07 compared to the remaining samples) and four patients with joint fluid samples (P=0.17 compared to the remaining samples). Taken together, 16SRS results from heart valve and joint fluid samples were more likely to result in a change in therapy than the other specimen sources (35% vs. 9.5%, p=0.01). Twenty-seven patients underwent MSCFP testing; 4 patients were immunocompromised, and 5 had culture-negative endocarditis. Of the 9 patients with immunodeficiency/endocarditis, results impacted care in 3 patients, versus 0 of 18 (P=0.01) remaining patients with other diagnoses, which included sepsis/fever of unknown origin (9 patients) and discitis (4 patients).

**Conclusion:**

16S ribosomal RNA had a greater impact on treatment decisions when the samples were from the heart valve or joint fluid. MSCFP more often impacted care in patients with severe immunodeficiency or culture-negative endocarditis. Laboratory stewardship guidelines should be developed to assist in the cost-effective utilization of molecular diagnostics in the general Infectious Diseases practice.

**Disclosures:**

All Authors: No reported disclosures

